# The Plasma Distribution of Non-cholesterol Sterol Precursors and Products of Cholesterol Synthesis and Phytosterols Depend on HDL Concentration

**DOI:** 10.3389/fnut.2022.723555

**Published:** 2022-03-01

**Authors:** Valéria Sutti Nunes, Eliton Juniro da Silva, Guilherme da Silva Ferreira, Sayonara Ivana Santos de Assis, Patrícia Miralda Cazita, Edna Regina Nakandakare, Vanessa Helena de Souza Zago, Eliana Cotta de Faria, Eder Carlos Rocha Quintão

**Affiliations:** ^1^Laboratorio de Lipides (LIM10), Hospital das Clinicas HCFMUSP, Faculdade de Medicina, Universidade de São Paulo, São Paulo, Brazil; ^2^Department of Clinical Pathology, School of Medical Sciences, State University of Campinas-UNICAMP, Campinas, Brazil

**Keywords:** 24-hydroxycholesterol, 27-hydroxycholesterol, desmosterol, lathosterol, campesterol, sitosterol, HDL

## Abstract

Non-cholesterol sterols are transported in plasma lipoproteins and are consequently important in cholesterol metabolism. We investigated the distribution of non-cholesterol sterol precursors of cholesterol synthesis (NCSPCS), oxysterols, and phytosterols in lipoproteins of healthy subjects differing according to HDL-Cholesterol (HDL-C) plasma levels. Elevated NCSPCS (desmosterol, lathosterol) in the High HDL group suggests that HDL exports these sterols from cells, but not the cholesterol metabolite 24-OHC which was higher in the Low HDL group than in the High HDL group. 27-hydroxycholesterol (27OH-C) plasma levels did not differ between groups. Percentage of NCSPCS and phytosterols predominates in LDL, but did not differ between groups. Thirty percent of desmosterol and lathosterol are present in HDL, with the High HDL group carrying higher percentage of these sterols. A high percentage of campesterol and sitosterol in HDL suggests that phytosterols are absorbed by enterocytes, and that HDL could be a marker of the ABCA1/ApoA1 intestinal activity.

## Introduction

Cholesterol synthesis rates like desmosterol, lathosterol, and squalene represent non-cholesterol sterol precursors of cholesterol synthesis (NCSPCS) that are carried by lipoproteins. Phytosterols, like campesterol and sitosterol, and also cholestanol (although being a cholesterol metabolite) reflect the efficiency of cholesterol absorption in hyperlipidemic populations (1–3).

Individuals with elevated high-density lipoprotein cholesterol (HDL-C) plasma levels have greater plasma concentrations of cholesterol absorption markers (campesterol and sitosterol) and lower plasma concentration of lathosterol which is a marker of body cholesterol synthesis (4).

Oxysterols are oxidized forms of cholesterol and also of its precursors that are formed in the first steps of cholesterol metabolism by the enzyme cytochrome P450s (CYP) (5). 7α-Hydroxycholesterol (7-OH-C) is formed from cholesterol by CYP7A1 and represents the first metabolite in the neutral pathway of bile acid biosynthesis (6, 7). 27-hydroxycholesterol (27-OHC), and 3β-hydroxycholest-5-en-(25R)26-oic acid (3β-HCA) are both formed from cholesterol by CYP27A1 and are the first members of the acidic, or alternative, pathway of bile acid biosynthesis (6, 8). CYP7A1 is an endoplasmic reticulum and liver specific protein. CYP27A1 is mitochondrial and expressed in many tissues (6). CYP46A1 is almost exclusively expressed in neurons, its function is to maintain cholesterol balance in the brain, converting cholesterol from a molecule unable to cross the blood-brain barrier to 24S-hydroxycholesterol (24S-OHC), a more polar molecule which crosses the barrier (9, 10). Recent data have shown oxysterols to be ligands to nuclear receptors and to G protein-coupled receptors, modulators of *N*-methyl-d-aspartate receptors, and regulators of cholesterol biosynthesis (6). Oxysterols are more than simple metabolites in the pathway from cholesterol to bile acids having anti-atherosclerotic activity by eliminating excess cell cholesterol through the ATP Binding Cassette Subfamily A Member 1 (ABCA1) (11), or by passive diffusion. As mandatory components that mediate cholesterol excretion, 24-hydroxycholesterol (24-OHC) and 27-hydroxycholesterol (27-OHC) are important molecules in maintaining body cholesterol homeostasis.

24-OHC, 27-OHC, and 3β-hydroxy-5-cholestenoic acid in lipoprotein fractions and lipoprotein-free plasma of seven healthy non-smoker volunteers indicate that 24-OHC and 27-OHC are similarly distributed in plasma lipoproteins (40% in LDL and 40–50% in HDL). The 24-OHC/cholesterol and the 27-OHC/cholesterol ratios were higher in the HDL-C fraction demonstrating the importance of HDL for carrying these oxysterols (12). In this regard, increased 27-OHC/cholesterol ratio in plasma of individuals with low HDL-C concentration compared to individuals with high HDL-C concentration suggests increased cellular cholesterol excretion via this pathway, thus protecting cells from cholesterol accumulation (13). Due to the renewed importance of HDL in the reverse cholesterol transport pathway (14, 15) the present study aimed at investigating the lipoprotein distribution of NCSPCS, phytosterols and oxysterols in healthy individuals that differ according to HDL-C plasma concentration.

## Materials and Methods

### Subjects

Volunteers of both genders were recruited from primary health care centers in Campinas (SP-Brazil) and Ambulatório de Dislipidemia do Serviço de Endocrinologia e Metabologia do Hospital das Clínicas da Faculdade de Medicina da Universidade de São Paulo (HCFMUSP).

The study was approved by the Research Ethics Committee of UNICAMP School of Medicine under n° 120/2007 and Hospital das Clínicas da Faculdade de Medicina da Universidade de São Paulo under n° 149/7. All participants were informed about the objectives of the protocol and signed a written consent according to research protocols approved by the Ethics Committee of HCFMUSP and UNICAMP.

The study included 20–74 years-old subjects, non-smokers, asymptomatic individuals with body mass index (BMI) lower than 30 kg/m2, without regular use of any medications that interfere with lipid metabolism and daily intake of alcohol lower than 14 g, as previously described (16).

Subjects were selected for having plasma HDL-C concentrations below 40 mg/dL (Low HDL group, 7 males, and 5 females), above 40 mg/dL and below 60 mg/dL (Control group, 4 males and 6 females), or above 60 mg/dL (High HDL group, 7 males, and 8 females). Low and high values were considered, respectively, below the 10th percentile and above the 90th percentile of the Brazilian population. The exclusion criteria were obesity, diabetes mellitus, metabolic syndrome, thyroid function disorders, liver and kidney failures, smoking, alcohol abuse, and use of medications that might interfere with the metabolism of cholesterol.

### Isolation of Plasma Lipoproteins

Blood from the Low HDL group, High HDL group and a Control group was drawn after 12 h fasting period into tubes containing ethylenediamine tetraacetic acid (EDTA) (10%). Plasma was immediately separated and added benzamidine 2 mM (5 μL/mL), gentamycin + chloramphenicol 15 mM (20 μL/mL), phenyllmethyl sulphonyl fluoride 0.5 mM (0.5 μL/mL) and aprotinin 10 mg/mL (5 μL/mL) and butylated hydroxytoluene (BHT). Plasma very-low-density lipoproteins (VLDL), low-density lipoproteins (LDL), high-density lipoproteins (HDL), and the lipid free fraction (LFF) were separated by sequential ultracentrifugation of plasma samples (10 mL) using a Beckman Model L-8 ultracentrifuge and 50 Ti rotor (Beckman Instruments, Palo Alto, CA, USA) (17). The plasma density was maintained at 1.006 to separate the VLDL fraction, obtained after 12 h ultracentrifugation, at 100,000 X g at 4°C. The infranadant of d>1.006 g/mL was adjusted with solid KBr to 1.063 g/mL to obtain the LDL fraction, after 20 h ultracentrifugation, at 100,000 X g and 4°C, and the plasma infranadant of d>1.063 g/mL was adjusted with solid KBr to 1.210 g/mL to obtain HDL after 40 h ultracentrifugation, at 100,000 X g and at 4°C.

### Biochemical Analysis

The measurements of total cholesterol, HDL-C, triglycerides and glucose were performed by conventional colorimetric enzymatic methods (Roche Diagnostics GmbH) in automated BM Hitachi 917 (Roche Diagnostics). The VLDL-C was estimated as a fifth part of triglyceridemia and LDL-C was calculated by Friedewald equation for triglyceridemia levels up to 400 mg/dl (18).

### Chromatographic Analysis

Plasma and lipoprotein total NCSPCS (desmosterol, lathosterol) and phytosterols (campesterol and sitosterol) were measured in samples (100 μL) added 5α-cholestane (1 μg) as the internal standard, hydrolyzed with KOH in ethanol (1 mol/l, 1 ml) at 60 °C (1 h) and extracted with hexane. Sterols were derivatized with a sylilating solution of pyridine and BSTFA (N,O-bis (trimethylsilyl) trifluoroacetamide) +1% TMCS (trimethylchlorosilane) (1:1, v/v) (Supelco 33155-U) for 1 h at 60°C (4). The derivatized sample (1 μL) was injected into a gas chromatograph coupled to a mass spectrometer (Shimadzu GCMS-QP2010, Kyoto, Japan). Efficient sterol separation was achieved in a Restek capillary column (100% dimethyl polysiloxane–Rxi13323) that was 30 m long, had a 0.25 mm internal diameter, contained helium as the mobile phase and had constant linear velocity of 45.8 cm/s with an oven temperature at 280°C. The mass spectrometer was operated in electron impact mode at an ionization voltage of 70 eV with a source temperature of 300°C for the ions and the interface. Single ion monitoring (SIM) was carried out by monitoring m/z = 109, 149 and 217 for 5α-cholestane, m/z =119, 253 and 351 for desmosterol, m/z = 213, 255 and 458 for lathosterol, m/z = 129, 343, and 382 for campesterol and m/z = 129, 357, and 486 for β-sitosterol enabling greater sensitivity in quantification. Quantification was based on the total ion chromatogram (total ion chromatogram, TIC) with correction by the internal standard 5α-cholestane and identification was based on comparison with the retention times and mass spectra of the standard curve.

Plasma and lipoprotein total oxysterols (24-OHC and 27-OHC) were measured according S. Dzeletovic et al. (19) as modified (20). Internal standard (100 ng of 24-hydroxycholesterol-d7 and 27-hydroxycholesterol-d7) in chloroform (Avanti Polar Lipids, Alabaster, USA) was added in 1 mL of plasma and 500 μL of lipoproteins. Oxysterols measurements were performed after alkaline hydrolysis adding a mixture of 10 mL of absolute ethanol and 0.4 M of potassium hydroxide overnight, at room temperature. The pH was adjusted to 7 with phosphoric acid followed by 20 mL of chloroform and 6 mL of water. After vigorous shaking and centrifugation at 4^0^C, the aqueous phase was removed and the organic phase evaporated. The lipid extract was dissolved in toluene (1 mL). Oxysterols were separated from cholesterol by solid phase extraction. Briefly, the sample was applied into the column (Sigma-Aldrich Supelclean LC-Si SPE Tubes SUPELCO, Bellefonte, USA) previously conditioned with hexane (8 mL). Cholesterol was eluted with 1.5% isopropanol in hexane (8 mL), and oxysterols were further eluted with 30% isopropanol in hexane (6 mL). Finally, the solvent was evaporated and samples were derivatized with a sylilating solution of pyridine and BSTFA +1% TMCS (1:1, v/v) (Supelco 33155-U) for 1 h at 60°C. The derivatized sample (1 μL) was injected into a gas chromatograph coupled to a mass spectrometer (Shimadzu GCMS-QP2010, Kyoto, Japan) by the automatic injector and analyzed in selected ion monitoring. The separation was performed on a Restek capillary column (100% dimethyl polysiloxane–Rxi13323), 30 m, internal diameter 0.25 mm, for 30 min, using helium as mobile phase, with constant linear velocity of 44.1 cm/s. The oven started at 240°C with increment of 5°C/min, for 7 min up to 290°C. The mass spectrometer was operated in electron impact mode at an ionization voltage of 70 eV with a source temperature of 300°C for the ions and the interface. Quantification was done in the SIM mode and the ions were monitored at m/z 145 and 129 for 24-hydroxicolesterol; m/z 151 and 129 for 24-hydroxycholesterol-d7; m/z 456 and 129 for 27-hydroxicolesterol; m/z 462 and 129 for and 27- hydroxycholesterol-d7. The quantification was performed comparing the peak areas of the standard curve and corrected for internal standards (20). Non-cholesterol sterols (μg) and oxysterols (ng) were expressed as their ratios to total cholesterol (mg), and to cholesterol belonging to each lipoprotein fraction.

### Statistical Analysis

The results were expressed as mean ± SD or the median (variation). Differences between groups were compared by Kruskal Wallis (*p* < 0.05) and Dunn's multiple comparison with correction by Bonferroni *post hoc* (*p* < 0.017) was performed when appropriate. Different letters represent statistically significant in the post-test. Gender distribution were compared by Chi-squared test (*p* < 0.05).

## Results

Differences in HDL-C plasma concentration of each group and characteristics of samples about anthropometric data, glucose, lipids, and lipoprotein concentrations in plasma are presented in Table 1. Triglycerides and VLDL-C plasma levels were lower in the High HDL group when compared to the Low HDL and Control groups. Triglycerides were within the normal range (<150 mg/dL) in all participants. To eliminate the influence of serum lipoprotein cholesterol concentration between individuals, all plasma sterols have been expressed in relation to plasma cholesterol (20, 21).

**Table 1:** Anthropometric data and plasma concentrations of glucose, lipids and lipoproteins.

	**Low HDL**	**High HDL**	**Control**	***p***
*n*	12	15	10	
Gender (men/woman)	7/5	7/8	4/6	
Age (years)	46 ± 14	52 ± 14	47 ± 11	
BMI (kg/m^2^)	24 ± 2	22 ± 2	23 ± 3	
Glucose (mg/dL)	89 ± 11	89 ± 7	89 ± 5	
Cholesterol (mg/dL)	163 ± 28	198 ± 37	173 ± 44	
LDL-C (mg/dL)	104 ± 17	108 ± 31	107 ± 39	
HDL-C (mg/dL)	34 (25–39) a	74 (62–95) b	46 (42–49) c	<0.001
VLDL-C (mg/dL)	21 ± 6 a	14 ± 6 b	22 ± 9 a	0.009
Triglycerides (mg/dL)	107 ± 30 a	71 ± 29 b	108 ± 44 a	0.011

*Results are expressed as the mean ± standard deviation (SD) or median (range). Kruskal Wallis (p < 0.05), Dunn's post-test with correction by Bonferroni. Different letters represent statistical significance in the post-test*.

*BMI, body mass index; VLDL-C, very-low-density lipoprotein cholesterol; LDL-C, low-density lipoprotein cholesterol; HDL-C, high-density lipoprotein cholesterol*.

The concentration of 24-OHC is higher in the Low HDL group (*p* = 0.024) when compared to High HDL participants but the percentage distribution in lipoproteins did not differ between groups (Table 2). The 27-OHC plasma levels and the percentage distribution in lipoproteins did not differ between the groups (Table 2) (*p* = 0.07).

**Table 2:** The 24-OHC and 27-OHC (ng) to cholesterol (mg) ratios in plasma and its percentage distribution among lipoproteins.

	**Low HDL**	**High HDL**	**Control**	***p***
*n*	12	15	10	
24-OHC plasma (ng/mg)	75 a (46–198)	52 b (34–100)	63 ab (9–120)	0.024
24-OHC percentage distribution (range)			
VLDL	14 (9–19)	10 (6–39)	20 (6–40)	
LDL	27 (16–70)	35 (14–49)	28 (14–54)	
HDL	32 (11–66)	35 (16–68)	38 (12–64)	
LFF	11 (0–45)	13 (3–37)	10 (6–31)	
27-OHC plasma (ng/mg)	113 (54–141)	86 (48–151)	123 (69–323)	0.07
27-OHC percentage distribution (range)			
VLDL	19 (14–47)	9 (0–26)	19 (0–35)	
LDL	36 (23–54)	36 (19–64)	41 (21–42)	
HDL	36 (30–50)	42 (28–64)	40 (26–60)	
LFF	0 (0–0)	0 (0–38)	0 (0–17)	

*Results are expressed as the median (range). Kruskal Wallis (P < 0.05), Dunn's post-test with correction by Bonferroni. Different letters represent statistical significance in the post-test*.

*VLDL, very-low-density lipoprotein; LDL, low-density lipoprotein; HD, high-density lipoprotein; LFF, lipoprotein free fraction*.

The NCSPCS (desmosterol and lathosterol) and phytosterols (campesterol and sitosterol) plasma values, expressed as μg/mg of cholesterol, did not differ among groups (Table 3). The percentage of NCSPCS and phytosterols predominated in LDL-C, which carries roughly 50% of these molecules (Table 3), but did not differ between groups. Approximately 30% of desmosterol and 25% of lathosterol were present in HDL, and the High HDL group had significantly higher percentage of these sterols compared to Low HDL and Control groups (Table 3). VLDL carried <20% of NCSPCS. The VLDL lathosterol percentage was lower in the High HDL group compared to the Low HDL and Control groups (Table 3). The High HDL group had significantly higher percent of campesterol and sitosterol compared to the Low HDL group. In contrast, higher sitosterol distribution occurred in the LDL fraction in the Low HDL group (Table 3).

**Table 3:** Desmosterol, lathosterol, campesterol, and sitosterol (μg) to cholesterol (mg) ratios in plasma and its percentage distribution among lipoproteins.

	**Low HDL**	**High HDL**	**Control**	***p***
*n*	12	15	9	
Desmosterol plasma (μg/mg)	0.770 (0.250–1.715)	0.633 (0.053–1.431)	0.508 (0.209–1.321)	
Desmosterol percentage distribution (range)			
VLDL	17 (9–39)	12 (0–38)	15 (3–23)	
LDL	54 (24–77)	55 (22–92)	64 (43–77)	
HDL	26 (7–40)	33 (8–64)	24 (14–40)	0.039
Lathosterol plasma (μg/mg)	0.188 (0.043–0.467)	0.159 (0.068–0.327)	0.272 (0.082–0.534)	
Lathosterol percentage distribution (range)			
VLDL	19 (11–55) a	14 (4–37) b	18 (11–31) ab	0.041
LDL	62 (29–76)	56 (17–69)	62 (49–68)	
HDL	16 (5–56) a	29 (20–59) b	20 (11–38) ab	0.006
Campesterol plasma (μg/mg)	1.437 (0.375–2.713)	1.066 (0.406–3.019)	0.933 (0.220–2.077)	
Campesterol percentage distribution (range)			
VLDL	20 (12–50)	15 (3–33)	17 (8–35)	
LDL	58 (37–70)	50 (22–59)	56 (47–63)	
HDL	21 (9–43) a	37 (26–51) b	28 (16–44) a,b	0.005
Sitosterol plasma (μg/mg)	1.244 (0.496–2.988)	1.402 (0.396–2.447)	0.949 (0.204–2.437)	
Sitosterol percentage distribution (range)			
VLDL	15 (10–51)	10 (2–65)	15 (9–35)	
LDL	60 (34–71) a	47 (21–55) b	51 (36–65) ab	0.028
HDL	23 (9–50) a	43 (11–67) b	33 (19–42) ab	0.002

*Results are expressed as the median (range). Kruskal Wallis (P < 0.05), Dunn's post-test with correction by Bonferroni. Different letters represent statistical significance in the post-test*.

*VLDL, very-low-density lipoproteins; LDL, low-density lipoproteins; HDL, high-density lipoproteins*.

**Figure 1:**
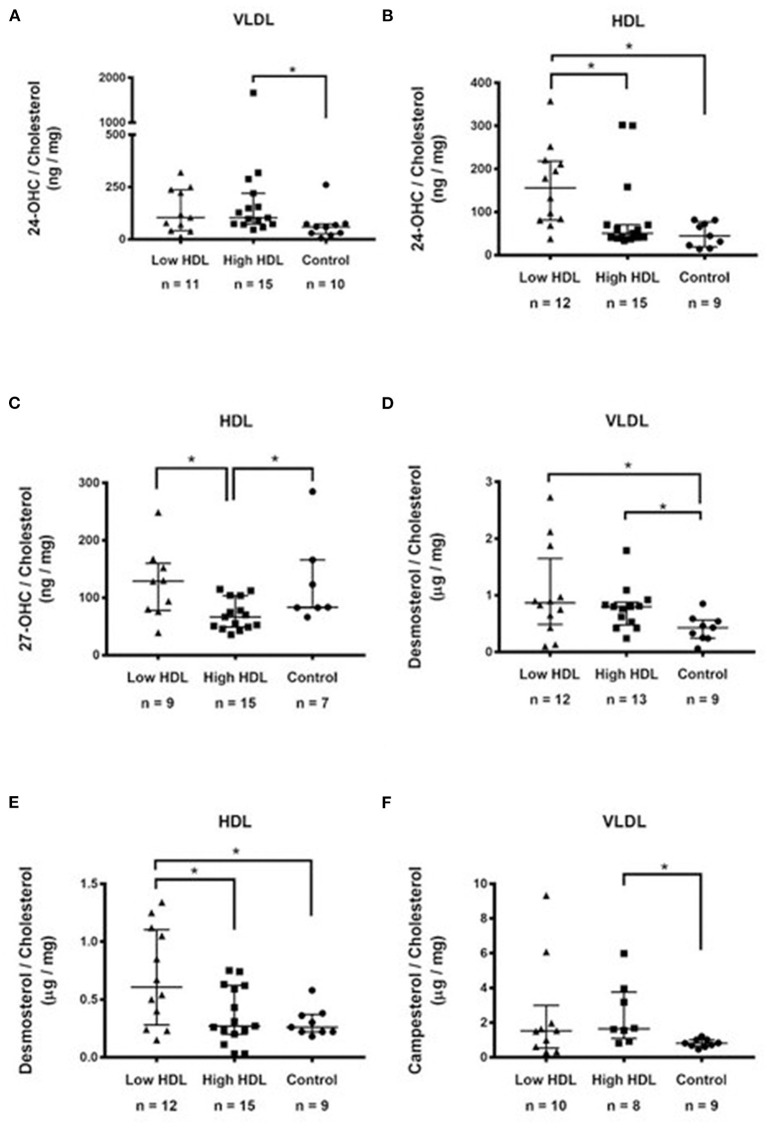
Sterols in lipoproteins expressed as their ratios to cholesterol belonging to each lipoprotein fraction: **(A)** 24-hydroxycholesterol (ng/mg) in VLDL; **(B)** 24-hydroxycholesterol (ng/mg) in HDL; **(C)** 27-hydroxycholesterol (ng/mg) in HDL; **(D)** Desmosterol in VLDL (μg/mg); **(E)** Desmosterol (μg/mg) in HDL; **(F)** Campesterol (μg/mg) in VLDL. Results were compared between groups by Kruskal-Wallis followed by the *post hoc* Dunn's test. *p < 0.05.

To better understand the biological significance of our results, we compared all sterol amounts amongst the three experimental groups by expressing them per lipoprotein particle as well as by cholesterol content in each lipoprotein component and only the statistically significant results are shown (Figure 1). As for 24-OHC in VLDL particles, the Control group had lower sterol contents than the HIGH HDL group (Figure 1A). As for 24-OHC in HDL particles, the LOW HDL group had higher sterol contents than others groups (Figure 1B). As for 27-OHC in HDL particles, HIGH HDL group had lower sterol amounts than the others group (Figure 1C). As for desmosterol in VLDL, it was higher in Low HDL and High HDL compared to Controls (Figure 1D). As for desmosterol in HDL particles, it was higher in Low HDL compared to High HDL and Controls (Figure 1E). Campesterol in VLDL was lower in the Control group compared to the High HDL (Figure 1F). Inversely proportional correlations were significant in HDL particles: 24-OHC/Cholesterol (ng/mg) vs. HDL-C (mg/dL), *r* = −0.35, *p* = 0.036, *n* = 36; 27-OHC/Cholesterol (ng/mg) vs. HDL-C (mg/dL), *r* = −0.53, *p* = 0.002, *n* = 31; Desmosterol/Cholesterol(ng/mg) vs.HDL-C (mg/dL), *r* = −0.32; *p* = 0.055, *n* = 36.

## Discussion

The distribution of oxysterols in plasma depends on their esterification rates by the enzyme lecithin-cholesterol acyltransferase (LCAT) (22), and their transfer rates between the lipoproteins mediated by the cholesterol transfer protein (CETP) (23). In this regard, LCAT activity was reported higher in individuals with low HDL-C plasma concentrations. The CETP activity did not differ between individuals with low and high plasma HDL-C levels (24).

We found lower 24-OHC (*p* = 0.024), and borderline lower 27-OHC (*p* = 0.07) plasma concentration (ng/mg of cholesterol) in the High HDL group (Table 2) even when expressed by the cholesterol content in each lipoprotein component (Figures 1B,C). Also, significant inversely proportional correlations were shown in HDL between 24-OHC/Cholesterol and 27-OHC/Cholesterol *vs*. HDL, suggesting that the High HDL plasma group does not provide greater efficiency for cellular export of cholesterol metabolites as previously shown (13).

There was no statistical difference in the percentage distribution of oxysterols amongst lipoproteins of the High HDL, Low HDL, and Control groups. The values are similar to those found in the literature describing the plasma concentration and distribution of these oxysterols among the lipoproteins of healthy individuals (12, 25). The human model in these studies is comparable to our Control group, namely, HDL-C between 40 and 60 mg/dL.

Similarly to cholesterol, NCSPCS and phytosterols are carried in lipoproteins (26). We found no differences among the groups in plasma NCSPCS lipoprotein concentrations.

Approximately 30% of desmosterol and lathosterol were present in HDL. VLDL transported <20% of NCSPCS. However, as for VLDL, the sterol composition seems to result from more complex simultaneous metabolic activities that directly involve their export by the liver and also faster rates of their exchange with higher density lipoprotein particles when expressed by cholesterol content in each lipoprotein component. Thus, the amount of 24-OHC, desmosterol and of campesterol in VLDL is lower in the Control group compared to the other groups (Figures 1A,D,F).

The High HDL group showed higher NCSPCS percent than the other groups. This means that although the fraction of these sterols transported in LDL was the largest, the increased participation of HDL suggests its role in the cellular removal of cholesterol synthesis precursors agreeing with previous work (27). Accordingly, Yamauchi et al. (27) showed that substantial amounts of precursor sterols are transported to a plasma membrane domain and are removed by the ABCA1-dependent pathway. However, when the results are expressed by particle cholesterol the opposite is obseved, i.e., the low HDL group has the highest desmosterol concentration, and significant inversely proportional correlations between desmosterol vs. HDL, whereas desmosterol in the VLDL particles was lower in the Control group compared to the other groups.

Resembling cholesterol, phytosterols, and phytostanols are carried in lipoproteins, being 70–80% in low density lipoprotein (LDL), and 20–30% in high density lipoprotein (HDL) (28, 29). In normal weight, good to moderate glucose balance, no insulin therapy, mild to moderate hypercholesterolemia, and normotriglyceridemia, in type 2 diabetics, campesterol and sitosterol concentrations were 7–9% in VLDL, 3–4% in IDL, 59–61% in LDL, and 27–30% in HDL (26). We found similar distributions of phytosterols as shown by Björkhem et al. (29), and Simonem et al. (26). On the other hand, in the PROCAM study (Prospective Cardiovascular Münster) patients with low HDL-C levels displayed decreased plasma phytosterol, and a direct correlation occurred between low HDL-C and decreased plasma phytosterol (30). However, PROCAM dealt with metabolic syndrome subjects, not with normal cases as in the present study.

Our High HDL group presents higher percent campesterol and sitosterol in HDL, suggesting that phytosterols absorbed by the enterocytes are incorporated into nascent HDL. Our data agree with the observations of higher plasma phytosterol levels in patients with high HDL-C levels matched for similar LDL-C levels (4), and in patients with high HDL-C levels due to exercise (31). However, this finding is not confirmed when expressing the campesterol and sitosterol for cholesterol concentration in lipoproteins.

It is worth noting that the increased phytosterol in cases of elevated HDL-C elicited by the CETP inhibitor Dalcetrapib was similar to that seen with statin treatment (32), and different from that measured in ABCG5/G8 mutation leading to atherogenic phytosterolemia (33). Based on these results it was proposed that phytosterols not returned to the intestinal lumen via ABCG5/G8 activity are absorbed via chylomicrons with trace amounts absorbed via an HDL pathway, and very likely are efficiently excreted by the liver (34). However, since measurable amounts of cholesterol are absorbed at the intestinal level through the ABCA1/ApoA1 system (35) it has been hypothesized that the absorption of phytosterol—which cannot be synthesized by animals—via the HDL pathway could be used as a marker of intestinal ABCA1/ApoA1 activity (35). According to Niesor et al. plant sterols offer the advantage of being only of dietary origin absorbed at the intestinal level via an HDL pathway (36), very likely due to ApoA1 lipidation with cholesterol, and consequently related to pre-beta-1 HDL levels (37). This observation (36) was made both in hamsters and healthy human volunteers treated with the CETP modulator Dalcetrapib, which affects HDL metabolism in both species.

In our study, increased percent of HDL campesterol and sitosterol in the High HDL group as compared to the other groups suggests phytosterols absorbed in the enterocytes mostly are incorporated into the nascent HDL, thus corroborating the hypothesis of Niesor et al. (35) that phytosterol absorption via the HDL pathway represents a marker of intestinal ABCA1/ApoA1 activity. An indirect proof of this concept is the lack of HDL-increase in plasma phytosterol on CETP inhibition with Dalcetrapib in patients with mutations in ApoA1 and/or ABCA1 (38).

## Conclusions

Elevated percentage HDL desmosterol and lathosterol in the High HDL group suggests HDL facilitates the export of these NCSPCS from cells but not the export of the cholesterol metabolite 24-OHC which was lower in the High HDL than in the Low HDL group. A high percentage of campesterol and sitosterol in the high HDL group suggests that phytosterols are absorbed by enterocytes, and incorporated mainly into nascent HDL corroborating the hypothesis that the phytosterol content in HDL could be a marker of the ABCA1/ApoA1 intestinal activity (35).

## Data Availability Statement

The raw data supporting the conclusions of this article will be made available by the authors, without undue reservation.

## Ethics Statement

The studies involving human participants were reviewed and approved by Research Ethics Committee of UNICAMP School of Medicine under n° 120/2007 Hospital das Clínicas da Faculdade de Medicina da Universidade de São Paulo under n° 149/7. The patients/participants provided their written informed consent to participate in this study.

## Author Contributions

VN: conceptualization, methodology, funding acquisition, and writing—review and editing. ES: investigation and writing—original draft. GF: formal analysis. SA: investigation. VZ: data curation. PC: investigation and writing—original draft. EN: writing—review and editing. EF: writing—review and editing and data curation. EQ: supervision and writing—review and editing. All authors contributed to the article and approved the submitted version.

## Funding

This research received financial support from Fundação de Amparo à Pesquisa do Estado de São Paulo, FAPESP (grants # 06/60585-9 to VN) and fellowship support to Eliton Junior da Silva (Grants # 2013/13631-9).

## Conflict of Interest

The authors declare that the research was conducted in the absence of any commercial or financial relationships that could be construed as a potential conflict of interest.

## Publisher's Note

All claims expressed in this article are solely those of the authors and do not necessarily represent those of their affiliated organizations, or those of the publisher, the editors and the reviewers. Any product that may be evaluated in this article, or claim that may be made by its manufacturer, is not guaranteed or endorsed by the publisher.
